# Development of targeted whole genome sequencing approaches for Crimean-Congo haemorrhagic fever virus (CCHFV)

**DOI:** 10.1016/j.virusres.2024.199464

**Published:** 2024-09-20

**Authors:** Jake D'Addiego, Sonal Shah, Ayşe Nur Pektaş, Bi̇nnur Köksal Bağci, Murtaza Öz, Sasha Sebastianelli, Nazif Elaldı, David J Allen, Roger Hewson

**Affiliations:** a London School of Hygiene and Tropical Medicine, Department of Infection Biology, Faculty of Infectious and Tropical Diseases, London, UK; b London School of Hygiene and Tropical Medicine, UK Public Health Rapid Support Team, Department of Infection Disease Epidemiology and Dynamics, Faculty of Epidemiology and Population Health, London, UK; c UK Health Security Agency, Virology and Pathogenesis Research Group, Salisbury, UK; d Sivas Cumhuriyet University, Cumhuriyet University Advanced Technology Application and Research Center (CUTAM), Sivas, Türkiye; e Sivas Cumhuriyet University, Department of Nutrition and Dietetics, Faculty of Health Sciences, Sivas, Türkiye; f Sivas Numune Hospital, Clinic of Infectious Diseases and Clinical Microbiology, Sivas, Türkiye; g Sivas Cumhuriyet University, Department of Infectious Diseases and Clinical Microbiology, Faculty of Medicine, Sivas, Türkiye; h Department of Comparative Biomedical Sciences, Section Infection and Immunity, School of Veterinary Medicine, Faculty of Health and Medical Sciences, University of Surrey, Guildford, UK

**Keywords:** CCHFV, Probe hybridisation capture, Next-generation sequencing, Targeted enrichment

## Abstract

Crimean-Congo haemorrhagic fever (CCHF) is the most prevalent human tick-borne viral disease, with a reported case fatality rate of 30 % or higher. The virus contains a tri-segmented, negative-sense RNA genome consisting of the small (S), medium (M) and large (L) segments encoding respectively the nucleoprotein (NP), the glycoproteins precursor (GPC) and the viral RNA-dependent RNA polymerase (RDRP). CCHFV is one of the most genetically diverse arboviruses, with seven distinct lineages named after the region they were first reported in and based on S segment phylogenetic analysis.

Due to the high genetic divergence of the virus, a single targeted tiling PCR strategy to enrich for viral nucleic acids prior to sequencing is difficult to develop, and previously we have developed and validated a tiling PCR enrichment method for the Europe 1 genetic lineage.

We have developed a targeted, probe hybridisation capture method and validated its performance on clinical as well as cell-cultured material of CCHFV from different genetic lineages, including Europe 1, Europe 2, Africa 2 and Africa 3. The method produced over 95 % reference coverages with at least 10x sequencing depth. While we were only able to recover a single complete genome sequence from the tested Europe 1 clinical samples with the capture hybridisation protocol, the data provides evidence of its applicability to different CCHFV genetic lineages.

CCHFV is an important tick-borne human pathogen with wide geographical distribution. Environmental as well as anthropogenic factors are causing increased CCHFV transmission. Development of strategies to recover CCHFV sequences from genetically diverse lineages of the virus is of paramount importance to monitor the presence of the virus in new areas, and in public health responses for CCHFV molecular surveillance to rapidly detect, diagnose and characterise currently circulating strains.

## Highlights

• A novel probe capture hybridisation method has been developed for CCHFV.
• The presented methodology was validated against clinical as well as cell-cultured reference viruses.
• The newly developed methodology produced near complete genome coverages at 10x sequencing depths.
• Successful recovery of genomes from different CCHFV genetic lineages was achieved.

## 1 Introduction

Crimean-Congo haemorrhagic fever (CCHF) is the most prevalent human tick-borne viral disease, with a reported case fatality rate of 30 % or higher (Hawman and Feldmann, 2023). The disease is caused by the CCHFV virus (CCHFV), an orthonairovirus within the *Nairoviridae* family (order *Bunyavirales*), and is endemic to vast geographical areas spanning Africa, Middle East, Asia and Eastern Europe (World Health Organization - WHO, 2022) and is emerging in Western Europe (Bernard et al., 2024; Lorenzo et al., 2023). The virus is commonly transmitted to humans by the bite of an infected tick, particularly ticks from the genus *Hyalomma* which are considered to be its most competent virus vector (Papa et al., 2017). Transmission through direct contact with viraemic bodily fluids in nosocomial settings or in abattoirs has also been reported (Bente et al., 2013). Due to the lack of licenced vaccines or effective therapeutics, CCHFV has been listed as a priority pathogen for accelerated research in the 2018 WHO R&D Blueprint (Mehand et al., 2018). Although a meta-analysis study has reported benefits in using the drug ribavirin during early stages of CCHFV infection (Arab-Bafrani et al., 2019), randomised trials are still lacking (Johnson et al., 2018), and in previous studies we have found no reduction in virus load or a mutagenic effect on virus genomes following ribavirin treatment (D'Addiego et al., 2023).

The virus contains a tri-segmented negative-sense RNA genome, comprising of small (S), medium (M) and large (L) segments which encode respectively the viral nucleoprotein (NP), the precursor to the viral glycoproteins (GPC) and the viral RNA-dependent RNA polymerase (Portillo et al., 2021). Whilst the S and L segments display 95 % or more amino acid conservation between strains, the M segment is much more diverse, with distantly related genetic lineages displaying <75 % amino acid conservation (Hawman and Feldmann, 2023).

CCHFV is one of the most genetically diverse arboviruses, with seven distinct genetic lineages traditionally named after the regions where the lineage was first reported and based on S segment sequences: Africa 1, Africa 2 and Africa 3; Asia 1 and Asia 2; Europe 1 and Europe 2 (Pickin et al., 2022), although the latter has recently been re-classified as Aigai virus (AIGV) (Papa et al., 2022). Phylogenetic analysis of the M and L segment sequences reveals the presence of nine and six lineages respectively, which are only partially consistent with the S segment-based lineages (Volynkina et al., 2022).

Simultaneous advances in both sequencing technologies – particularly second and third-generation platforms by Illumina and Oxford Nanopore Technologies – and in accessible high-performance computing solutions, have advanced approaches for determining virus genome sequences using targeted and non-targeted methods.

Amplicon based, targeted approaches are based on polymerase chain reaction (PCR) amplification which has high-specificity, relatively low cost, and straightforward assay design (Houldcroft et al., 2017). Further, PCR-based targeted methods can be developed as multiplex tiling approaches, which have been successful for several viral pathogens of human and veterinary importance including tick-borne encephalitis virus (TBEV) (Zakotnik et al., 2022), Zika virus (ZIKV) (Quick et al., 2017), respiratory syncytial virus (RSV) A and B (Maloney et al., 2022), Bagaza virus (BAGV) (Sekee et al., 2024) and infectious bronchitis virus (IBV) (Le et al., 2024).

Recently, we reported recovery of near complete CCHFV genome sequences from sera collected from CCHF patients with low viral loads (< 10^6^ copies/ml of serum), for which a non-targeted approach failed to produce sufficient reads or coverage for consensus calling (D'Addiego et al., 2024), thereby demonstrating that targeted approaches are preferable over non-targeted sequencing approaches which are hindered by a lack of sensitivity in retrieving whole genome sequences. Despite the benefits of increased sensitivity, a major limitation of amplicon-based approaches is that their stringent specificity can make these methods unsuitable for highly genetically diverse viruses such as CCHFV, as such viruses would require multiple amplicon schemes tailored to different genetic lineages.

As an alternative, hybridisation probe capture techniques utilise DNA or RNA probes, referred to as “baits”, which are specifically designed to be complementary to the target genomes of interest. This method is adept at accommodating genetic diversity. By integrating comprehensive sequence data into the probe design process, baits can be optimised to cover a broad spectrum of genetic variations within the virus population. Furthermore, the increased length of probes compared to traditional PCR primers offers a higher degree of tolerance to mismatches, allowing for effective hybridisation despite minor sequence mismatches. This enhanced adaptability makes the baits approach highly suitable for genetically diverse viruses and complex sample types (Nasir et al., 2020; Tisza et al., 2023). Despite improvements in reference coverages have been described compared to non-targeted enrichment protocols for samples with low virus load, challenges remain in recovering full genome sequences from genetically divergent viruses, for which only conserved regions may be recovered (Wylezich et al., 2021).

The fast mutation rate of RNA viruses (Damian et al., 2020) such as CCHFV, the geographical expansion of competent vectors driven by climatic factors (Estrada-Pena, 2023) coupled with anthropogenic factors such as fragmentation of agricultural land, trade in livestock and religious festivals play a role in increased CCHFV transmission (Estrada-Pena et al., 2007), making the investigation of CCHFV a public health priority. Development of strategies to recover CCHFV sequences from genetically diverse lineages of the virus is of paramount importance to monitor the presence of the virus in new areas, and in public health responses for CCHFV molecular surveillance to rapidly detect, diagnose and characterise currently circulating strains.

The aim of this study was to validate a novel set of hybridisation probes for capture of CCHFV RNA, using cell-cultured CCHFV isolates as well as RNA from primary clinical samples, using short-read (Illumina) sequencing. We evaluate the performance of the hybridisation probes against our previously published CCHF Europe I tiling PCR scheme (D'Addiego et al., 2024) in a lineage-specific manner, as well as demonstrating that the baits can capture CCHFV RNA from multiple distinct genetic lineages.

## 2 Materials and methods

### 2.1 Reference CCHFV strains

CCHFV from genetic clades Africa 2, Africa 3, Europe 1 and Europe 2/AIGV were cultured at containment level (CL) 4 at UK Health Security Agency (UKHSA). Viruses were inoculated into SW13 cells, and infections allowed to proceed for up to 3 days, after which viral RNA was purified as described below.

### 2.2 Clinical sample collection

Following admission, acute stage (within 7 days from patients’ symptoms onset) serum samples were collected from 18 adult patients (18 years and over) admitted to Sivas Cumhuriyet University Hospital (SCUH), clinical service of Infectious Diseases and Clinical Microbiology, Sivas, Türkiye, between May and August 2020. CCHF diagnosis was based on a positive commercial CCHFV-specific real-time reverse-transcriptase polymerase chain reaction (RT-PCR) test (RealStar® CCHFV RT-PCR Kit 1.0, Altona Diagnostics) result for patients presenting with symptoms of viral haemorrhagic fever (VHF) infection. Samples were immediately stored at −80 °C until further processed. The study was approved by the local Ethics Committee of Sivas Cumhuriyet University (Protocol # 2019–09/04) and by the LSHTM Ethics Committee (LEO 28,159). All patients provided an informed consent form.

### 2.3 Sample processing and RNA extraction

Serum samples and cell-cultured viruses were inactivated in a CL4 laboratory; 140 µl of each sample were added to 560 µl of buffer AVL (QIAGEN) and 560 µl of molecular grade ethanol (Fisher Scientific) as previously described (Burton et al., 2017). RNA was purified using the QIAamp Viral RNA Mini Kit (QIAGEN) following the manufacturer's instructions. RNA was eluted in 60 µl of buffer AVE (QIAGEN) and stored at −80 °C.

### 2.4 Real-time reverse-transcription PCR

All purified RNAs were quantified in duplicate utilising an in-house real-time RT-PCR assay as previously described (D'Addiego et al., 2023).

Copy number of CCHFV genomic RNA were determined based on standard curves of a synthetic RNA in-vitro transcript (IVT) designed against a Europe 1 CCHFV S segment (accession number AY277672.1). Quantitative real-time RT-PCR was performed on a QuantStudio 7 Flex Real-Time PCR System (Applied Biosystems) platform with the following cycling parameters: 50 °C for 10 minutes, 95 °C for two minutes and 45 cycles of 95 °C for 10 seconds and 60 °C for 30 seconds.

### 2.5 Illumina library preparation

#### 2.5.1 CCHFV tiling multiplex PCR

cDNA synthesis and multiplex PCR enrichment were carried out as previously described (D'Addiego et al., 2024). Briefly, cDNA was synthesised using SuperScript™ IV First-Strand Synthesis System (Thermo Fisher Scientific) in final 20 µl reactions. Target enrichment was performed using two separate primer pools for each CCHFV genome segment as previously described (D'Addiego et al., 2024). Amplification occurred in triplicate 25 µl reactions using 2.5 µl of cDNA and containing one of two sets of primer pools utilising Q5® High-Fidelity 2X Master Mix (New England Biolabs [NEB]). Amplification occurred in a thermocycler with the following cycling parameters: 98 °C for 30 seconds, 30 cycles of 98 °C for 15 seconds, 57 °C for 30 seconds and 70 °C for 30 seconds.

The NEBNext® Ultra™ II FS DNA Library Prep Kit (NEB) was used for library preparation according to the manufacturers Illumina protocol for input DNA lower than 100 ng. Each individual 500 base pairs (bp) amplicon pool was adjusted to a final concentration of 0.4 ng/µl and made up to 26 µl using molecular grade water. Subsequently, fragmentation and end preparation were performed by incubating the amplicons at 37 °C for five minutes. Adaptors were diluted to 1.5 µM prior to ligation with Ultra II ligation master mix and Ligation enhancer supplied with the kit. USER enzyme, also supplied with the kit, was added. Post ligation, samples were cleaned up using AMPure XP beads (Beckman Coulter) and eluted in 17 µl of 0.1X TE buffer as described in the protocol. Fifteen microlitres of library were then utilised as input for PCR reactions. PCR reactions occurred in total 50 µl volumes using the Q5 polymerase (NEB) with the following cycling conditions: initial denaturation step at 98 °C for 30 s, followed by 13 cycles of a denaturation step at 98 °C for 10 s and an annealing/extension step at 65 °C for 75 s, followed by a final extension step at 65 °C for five minutes. PCR amplified libraries were cleaned up using AMPure XP beads and eluted in 33 µl of TE buffer (Invitrogen).

#### 2.5.2 CCHFV cDNA

Illumina libraries for hybridisation capture enrichment were prepared following the NEBNext® Ultra™ II FS DNA Library Prep Kit for Illumina (NEB) protocol (Input > 100 ng), according to the manufacturer's instructions. In brief, all sample volumes were adjusted to 26 µl volumes with molecular grade water (Thermo Fisher Scientific). Fragmentation/End preparation was performed by incubating samples at 37 °C for 10 min. Subsequently, adaptor ligation was performed as per the protocol with USER enzyme. Following adaptor ligation, size selection was performed with two supplied SPRI beads clean-ups as indicated in the protocol for insert sizes of 150–250 bp. The libraries were then amplified by PCR following the protocol for combined forward and reverse primers with 13 cycles as done for the amplicon-enriched samples. Following PCR, the libraries were pooled prior to clean-up into three pools as follows; two pools contained nine clinical samples and one of the negative template controls, and one pool contained the five cDNA samples from cell-cultured viruses. AMPure XP beads were used to clean-up the three library pools using 0.9X beads to sample volumes and eluted in 17 µl of 0.1X TE buffer. One microlitre of each sample was then quantified with Qubit dsDNA HS assay, and 12 µl of clean pooled library were then taken to the hybridisation step.

### 2.6 Custom CCHFV probe panel design

All CCHFV sequences were sourced from the National Centre for Biotechnology Information (NCBI) GenBank and curated. This curation process ensured that sequence orientation aligned with the open reading frame (ORF). Subsequently, sequences below 500 bp in length, those with extensive degenerate bases indicating low quality, and duplicate sequences were excluded. This resulted in a refined dataset comprising a total of 714 CCHFV sequences. A proprietary algorithm was used to generate and synthesise 6511 unique bait sequences of 120 bp length, complementary to and spanning the length of each genome sequence in the refined dataset by Agilent.

### 2.7 Hybridisation capture target enrichment

Hybridisation capture was performed utilising the SureSelect XT HS and XT Low Input Target Enrichment kit (Agilent) following the manufacturer's protocol. Probes were utilised at a 1:10 dilution per sample in each pool. Hybridisation mixes were prepared according to the manufacturer's instructions for probes < 3Mb. The library was incubated with the blocking solution prior to hybridisation to the probes for 60 min. During the hybridisation incubation the Dynabeads™ MyOne™ Streptavidin T1 streptavidin beads (Thermo Fisher Scientific) were washed with 200 µl of SureSelect binding buffer as described in the manufacturer's protocol for a total of three washes, and then re-suspended in fresh binding buffer.

Each hybridised library was added to 200 µl of resuspended and washed streptavidin beads, and capture was performed by mixing at 1400 rpm at room temperature for 30 min. Following capture, streptavidin-bound probes were washed as described in the manufacturer's protocol in wash buffer 1 followed by 6 washes in pre-heated (70 °C) wash buffer 2. Samples were eluted in 25 µl of molecular grade water. PCR was performed as described in the protocol with 24 cycles in total 50 µl reactions. Samples were cleaned up with SPRI beads at a 1:1 PCR reaction to beads ratio. Libraries were cleaned with 200 µl of freshly prepared 80 % ethanol and eluted in 25 µl of nuclease-free water.

### 2.8 Illumina sequencing

All libraries were quantified using the Qubit dsDNA HS assay (Thermo Fisher Scientific) and Bioanalyzer HS DNA assay (Agilent) and normalised to 4 nM with molecular grade water. Libraries were pooled and the final library was denatured in freshly made 0.2 N NaOH for five minutes and diluted to final 8 pM concentration using HT1 buffer supplied with the MiSeq Reagent Kit v2 Micro (Illumina). Libraries were sequenced on the Illumina MiSeq on a paired-end 150 bp run.

### 2.9 Bioinformatic analysis

Illumina FASTQ files were processed with Trimmomatic (Bolger et al., 2014) (version 0.39) as previously described (D'Addiego et al., 2024). Reads were mapped against CCHFV Europe I reference sequences (GQ337053-GQ337055 for the S, M and L segments respectively) using BWA-MEM (Li and Durbin, 2009) algorithm (version 0.7.17-r1188). Accession numbers for reference sequences from other CCHFV genetic lineages were KY484034-KY484036 (Africa 3); DQ076412, DQ094832 and DQ076413 (Africa 2); MK299343-MK299341 (Europe 2) for the L, M and S segments respectively.

Reference coverage and sequencing depth statistics were derived using SAMtools (Danecek et al., 2021) depth, coverage and flagstat functions. Consensus sequences were derived from sorted BAM files using BCFtools (Danecek et al., 2021) masking regions below 10x (for tiling PCR) or 1x (for probe hybridisation capture). For the tiling PCR data, amplicon primer sequences were removed from sorted BAM files using BAMClipper (Au et al., 2017) prior to consensus calling. Generated consensus sequences have been deposited in GenBank with the following accession numbers: PP735307-PP735360 (tiling PCR) and PP735361-PP735375 (hybridisation capture).

### 2.10 Phylogenetic analysis

Phylogenetic analysis was performed using MEGA 7 (Kumar et al., 2016) software. All near complete (> 90 % coverage) genome sequences generated in the study were included in the analysis. Sequences were aligned using MUSCLE (Edgar, 2004). Sequence Alignments were trimmed to a final length of 1504 bp (S), 3056 bp (M) and 5062 bp (L) with gaps prior to phylogenetic tree generation. Evolutionary history was inferred using the Maximum Likelihood method based on the General Time Reversible model. Bootstrap support values were generated with 1000 replicates.

## 3 Results

### 3.1 Tested samples presented with a range of viral RNA copies/ml

**Fig. 1:**
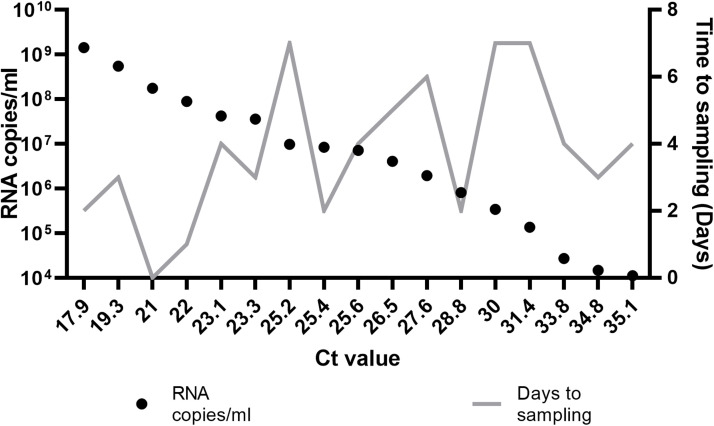
RNA copies/ml of serum (black dots) and time to sampling (days, grey line) from patients' symptoms onset.

The tested clinical samples presented with a range of RNA copies/ml of serum from 1.11 × 10^4^ to 1.43 × 10^9^, with corresponding Ct values ranging between 17.9 and 35.1 (Fig. 1). One sample yielded an undetermined RT-PCR result and was excluded from downstream analysis.

The cell-cultured viruses had RNA copies/ml of cell culture ranging between 3.73 × 10^5^ and 2.68 × 10^6^, with Ct values ranging between 24.12 and 26.97 (Supplementary Table 1).

### 3.2 Europe 1 lineage tiling PCR enrichment produced a greater proportion of mapped sequencing reads compared to the probe hybridisation capture protocol

**Fig. 2:**
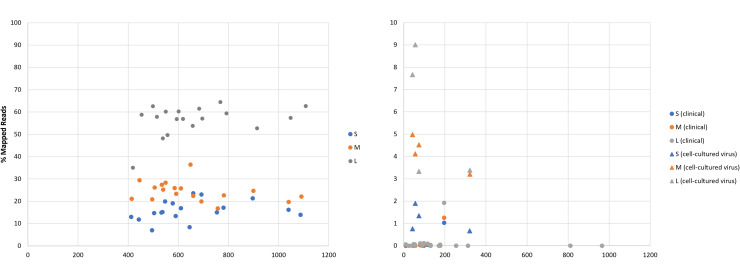
Percentage of mapped sequencing reads over total reads (thousands) for (A) tiling PCR enrichment and (B) probe hybridization capture. Genetic segments are colour-coded in blue (S), orange (M) and grey (L). Circular markers represent clinical samples; triangular markers represent cell-cultured viruses.

The average number of reads generated from 18 clinical samples using the tiling PCR approach across the three genome segments was 660,224 (range: 414,760–1,095,480; Fig. 2A). The percentage of reads mapping against the reference genome segment sequences were 15.81 % for the S segment (range 6.98 %–23.6 %), 24.34 % for the M segment (range 16.81 %–36.44 %) and 56.43 % for the L segment (range 35.03 %–64.48 %) respectively. The overall average percentage of mapped sequencing reads generated by tiling PCR was 32.19 %.

The average number of reads generated from the same clinical samples across the three genome segments using probe hybridisation capture was 201,911 (range 9532–965,598; Fig. 2B). The average percentage of mapped sequencing reads was 0.07 % for the S segment (range 0 %–1.03 %), 0.09 % for the M segment (range 0 %–1.25 %) and 0.14 % for the L segment (range 0 %–1.92 %). The overall average percentage of mapped sequencing reads generated by probe hybridisation capture was 0.1 %.

An average of 123,078 sequencing reads were produced from four cell-cultured virus samples using probe hybridisation capture (range 41,781 – 321,566, Fig. 2B), of which on average 1.17 % mapped against the S segment (range 0.76 %–1.9 %), 4.21 % against the M segment (range 3.21 %–4.98 %) and 5.85 % against the L segment (range 3.33 %–9.01 %) reference sequences.

### 3.3 Near complete reference coverage was produced from clinical samples with both tiling PCR and probe capture hybridisation approaches

**Fig. 3:**
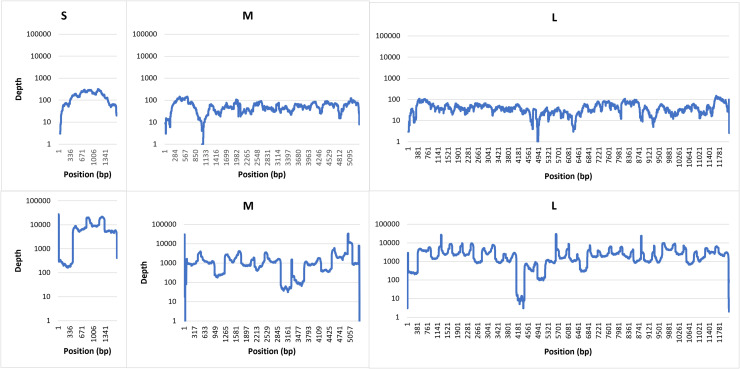
Reference coverage and sequencing depth for (A) probe hybridisation capture and (B) tiling PCR enrichment with Illumina sequencing technology on a CCHFV Europe 1 positive clinical serum sample (sample 4).

Coverages of 97.25 %, 99.94 % and 100 % were achieved for the S, M and L segments respectively for a CCHFV Europe 1 clinical sample (Sample 4; 1.75 × 10^8^ copies/ml) utilising the developed probe capture hybridisation method (Fig. 3A). With the tiling PCR approach, coverages of 100 %, 99.24 % and 94.32 % were achieved for the S, M and L segments respectively (Fig. 3B).

The mean sequencing depths using the probe capture hybridisation approach were 44.42x, 27.38x and 21.12x for the S, M and L segments respectively (Fig. 3A). The mean sequencing depths utilising the tiling PCR approach were 7510.48x, 1726.67x and 2617.56x for the S, M and L segments respectively (Fig. 2B).

### 3.4 Probe hybridisation capture produced coverages above 95 % at 10x sequencing depth

**Fig. 4:**
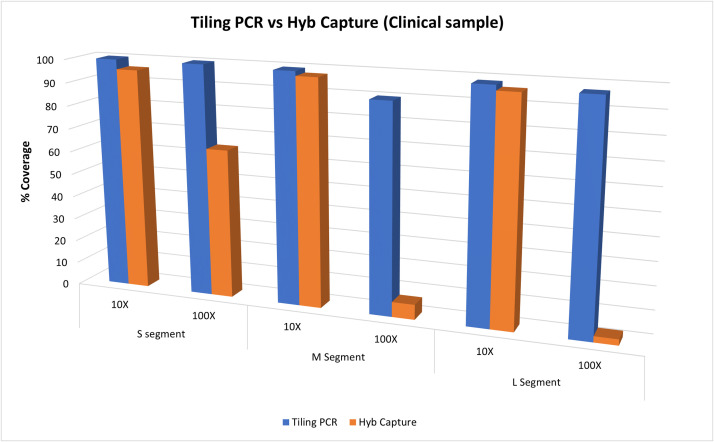
Reference coverages (%) for the S, M and L segments at 10X and 100X sequencing depths for tiling PCR (blue) and hybridisation capture (orange) on a CCHFV Europe 1 positive clinical sample (sample 4).

The tiling PCR approach produced at sequencing depths of 10x and 100x coverages of 100 % for the S segment. Equivalent metrics for the M segment were 99.37 % and 89.82 %, while for the L segment 98.43 % and 97.09 % (Fig. 4 blue columns). Coverages of 76.27 %, 48.79 % and 75.06 % were achieved at 1000x sequencing depths for the S, M and L segments respectively (data not shown).

With the hybridisation capture method, coverages at 10x and 100x sequencing depths were 95.88 % and 64.38 % for the S segment, 97.41 % and 6.47 % for the M segment and 96.21 % and 2.59 % for the L segment (Fig. 4 orange columns). Coverages at 1000x sequencing depth were not produced with the hybridisation capture method.

### 3.5 Probe hybridisation methodology yields near-complete genome sequences from four different CCHFV genetic lineages

Using the developed probe hybridisation capture protocol, reference coverages ranging between 94.24 %−99.24 %, 83.41 %−100 % and 90.19 %−100 % were achieved for the S, M and L segments respectively for the cultured CCHFV material from genetic lineages Europe 1 (Kosovo 2009), Africa 3 (IbAr10200), Africa 2 (Semunya) and Europe 2 (AP92) (Table 1). Average sequencing depths and mapped sequencing reads are presented in Table 1. Reference coverages at 10X and 100X ranged between 80.91 %−98.80 % and 0 %−81.59 % respectively for the S segment, between 76.98 %−99.29 % and 6.47 %−90.18 % respectively for the M segment and between 70.32 %−99.47 % and 0.92 %−60.40 % respectively from the L segment.

**Table 1:** Reference coverages (%), average sequencing depths and mapped sequencing reads for the S, M and L segments of cell-cultured CCHFV lineages Europe 1 (Kosovo 2009), Africa 3 (IbAr10200), Africa 2 (Semunya) and Europe 2 (AP92).

Lineage	Coverage (%)			Avg. depth			Mapped Reads		
	S	M	L	S	M	L	S	M	L
Europe 1	98.15	99.68	90.19	75.95	73.03	23.38	1040.00	3312.00	2441.00
Africa 3	94.73	93.85	93.48	23.98	47.09	32.56	317.00	2088.00	3219.00
Africa 2	99.24	100.00	100.00	182.77	237.83	118.59	2308.00	10,314.00	11,628.00
Europe 2	88.75	99.63	99.63	79.37	48.60	50.22	1056.00	2293.00	5040.00

**Fig. 5:**
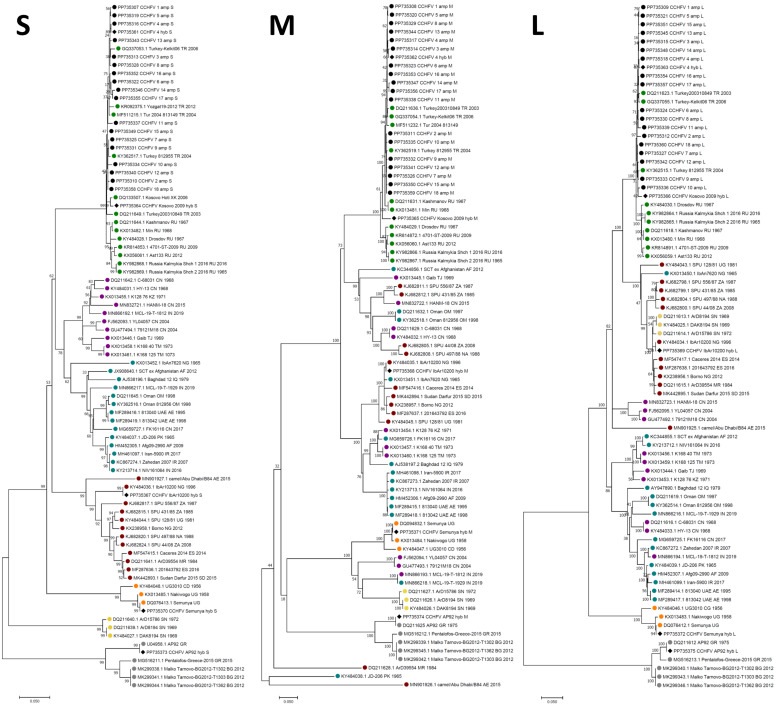
Maximum Likelihood phylogeny of the S, M and L CCHFV partial genome segments. Enrichment strategy in the generated consensus name is indicated by “amp” and “hyb” for the tiling PCR and capture hybridisation respectively.

The generated consensus sequences from the cell-cultured CCHFV strains clustered by phylogenetic analysis with their corresponding previously published sequences where one was available within the Europe 2 (AP92), Africa 2 (Semunya) and Africa 3 (IbAr10200) genetic lineages for all segments (Fig. 5). Consensus sequences for the cell-cultured Kosovo 2009 strain as well as the processed clinical samples for the S, M and L segments clustered within the Europe 1 lineage by phylogenetic analysis.

## Discussion

The northward range expansion of many tick species brought by climate change will likely expand the geographical range of many tick-borne viruses such as CCHFV, presenting public health laboratories involved in active virus surveillance with novel challenges. The potential introduction of viruses into new geographic locations, coupled with the fast-evolving nature of RNA viruses will require existing molecular diagnostics such as RT-PCRs to be continuously assessed and optimised against currently circulating viral strains, as well as novel diagnostic tools to be introduced against emerging viral pathogens. Targeted and metagenomic sequencing approaches will increasingly play an important role to genetically characterise circulating viruses and serve as agnostic diagnostic tools.

In this study, we presented a novel probe hybridisation capture protocol to recover near complete CCHFV genome sequences and validated its performance on clinical as well as cell-cultured CCHFV strains. The methodology was run alongside the previously published Europe 1 specific tiling PCR method (D'Addiego et al., 2024).

Similar to the study by Nasir et al. (2020) which compared different library preparation methodologies for two strains of SARS-CoV-2, we also observed a greater amount of sequencing reads produced using the tiling PCR approach compared to the probe hybridisation capture methodology. This outcome is expected due to the exponential amplification of viral target by PCR in the former approach. While Nasir et al. found that most of the sequencing reads produced after probe capture mapped against their Iran SARS-CoV-2 reference (but not for the Wuhan strain) using a random hexamers only protocol, our study revealed the majority of our sequencing reads did not map against our viral reference following hybridisation capture (Fig. 1B). This suggests the possibility of off-target genetic material still present in the sequencing libraries post probe capture. The variation in mapped sequencing reads between our study and Nasir's study is likely due to differences in sample types, patients’ variability, levels of non-viral host/background nucleic acids and variations in sample handling during collection, testing and storage. These factors are known to influence sequencing output (Kafetzopoulou et al., 2018). In contrast, the tiling PCR approach yielded a significant number of target viral reads (Fig. 1A), consistent with the findings of our previous study (D'Addiego et al., 2024). These higher sequencing depths enhance confidence in consensus calling.

Whist the tiling PCR method allowed us to obtain near complete genome sequences for all tested clinical samples, the probe hybridisation capture method only yielded a single complete CCHFV genome sequence (Fig. 2A). This difference could be attributed to viral RNA degradation during transit from Türkiye, or during long-term sample storage (Relova et al., 2018), indicating that for these sample types, a tiling PCR approach may be beneficial after initial genetic characterisation to recover complete genome sequences. Although the sequencing depths for the hybridisation capture methodology were significantly lower (on average 169.08-fold, 63.06-fold and 123.94-fold for the S, M and L segments respectively), good level reference coverages were achieved at a 10x sequencing depth (Fig. 3) not only for the clinical sample, but also for cell-cultured material from different CCHFV genetic lineages (including Europe 1, Europe 2, Africa 2 and Africa 3; Table 1).

The affiliation of all consensus sequences generated for the Turkish clinical samples within the Europe 1 lineage is expected, with all generated consensus sequences clustering most closely with previously published Turkish sequences by phylogenetic analysis for all genomic segments (Fig. 4). The clustering of consensus sequences from the cell-cultured viruses with their corresponding previously published sequences (Fig. 4) further validates the accuracy of the presented methodology in recovering viral genome sequences.

As new CCHFV sequences are published in public repositories, future work will focus on optimising the probe capture hybridisation method, which is currently limited by the number of publicly available sequences. This will involve designing additional probes targeting CCHFV genomes from genetic lineages that are currently still under-represented. Furthermore, additional steps in the sequencing library preparation such as DNase treatment prior to cDNA synthesis as well as the utilisation of specific primers during cDNA synthesis will also be tested. The optimisation aims to increase the sequencing depth and sensitivity of the hybridisation capture method. The performance of the presented methodology will also be evaluated across samples with different virus titres to determine its sensitivity, additional samples from different CCHFV genetic lineages, and other sample types such as tick homogenates.

Due to the high genetic diversity of CCHFVs, developing a single tiling PCR method to target all genetic lineages is challenging. The presented methodology could be utilised to recover sufficient genetic information for initial virus characterisation in CCHFV endemic regions in which circulating viruses have not yet been genetically characterised or their presence is still unknown. Following initial genetic characterisation of viruses, a targeted, lineage-specific tiling PCR method could be designed and used to recover complete genome sequences.

## CRediT authorship contribution statement

**Jake D'Addiego:** Writing – review & editing, Writing – original draft, Methodology, Formal analysis, Data curation. **Sonal Shah:** Writing – review & editing, Methodology, Formal analysis, Data curation. **Ayşe Nur Pektaş:** Resources. **Bi̇nnur Köksal Bağci:** Resources. **Murtaza Öz:** Resources. **Sasha Sebastianelli:** Methodology. **Nazif Elaldı:** Resources, Funding acquisition, Data curation. **David J Allen:** Writing – review & editing, Supervision, Resources, Project administration, Methodology, Funding acquisition, Formal analysis, Data curation, Conceptualization. **Roger Hewson:** Writing – review & editing, Supervision, Resources, Project administration, Methodology, Funding acquisition, Data curation, Conceptualization.

## Declaration of competing interest

The authors declare that they have no known competing financial interests or personal relationships that could have appeared to influence the work reported in this paper.

## Data Availability

Data will be made available on request.

## Appendix Supplementary materials

Image, application 1
