# Supplementary material for: Development of targeted whole genome sequencing approaches for Crimean-Congo haemorrhagic fever virus (CCHFV)
Source: Virus Res. 2024 Sep 20;350:199464. doi: 10.1016/j.virusres.2024.199464 (PMC11439567; doi:10.1016/j.virusres.2024.199464)
Supplement: Supplementary file 1 [file mmc1.docx]

**Supplementary Table 1 –** Ct values and RNA copies/ml for clinical and cell-culture virus samples.

| **Clinical** | | |  |  |
| --- | --- | --- | --- | --- |
| Sample ID | Ct value | RNA copies/ml serum |  |  |
| 1 | 31.4 | 1.36 x 10^5^ |  |  |
| 2 | 19.3 | 5.47 x 10^8^ |  |  |
| 3 | 33.8 | 2.71 x 10^4^ |  |  |
| 4 | 21 | 1.75 x 10^8^ |  |  |
| 5 | 35.1 | 1.11 x 10^4^ |  |  |
| 6 | 25.4 | 8.39 x 10^6^ |  |  |
| 7 | 34.8 | 1.47 x 10^4^ |  |  |
| 8 | 23.3 | 3.55 x 10^7^ |  |  |
| 9 | 22 | 8.82 x 10^7^ |  |  |
| 10 | 30 | 3.41 x 10^5^ |  |  |
| 11 | 17.9 | 1.43 x 10^9^ |  |  |
| 12 | Undetermined | Undetermined |  |  |
| 13 | 25.2 | 9.73 x 10^6^ |  |  |
| 14 | 26.5 | 4.04 x 10^6^ |  |  |
| 15 | 27.6 | 1.94 x 10^6^ |  |  |
| 16 | 23.1 | 4.18 x 10^7^ |  |  |
| 17 | 28.8 | 8.10 x 10^5^ |  |  |
| 18 | 25.6 | 7.15 x 10^6^ |  |  |
| **Cell-cultured virus** | | |  |  |
| Sample ID | Ct value* | RNA copies/ml of cell culture |  |  |
| Kosovo 2009 (Europe 1) | 24.12 | 2.68 x 10^6^ |  |  |
| IbAr10200 (Africa 3) | 26.97 | 3.73 x 10^5^ |  |  |
| Semunya (Africa 2) | 24.85 | 1.62 x 10^6^ |  |  |
| AP92 (Europe 2) | 25.77 | 8.61 x 10^5^ |  |  |

*Ct values have been adjusted to reflect the dilution factor used in this research.
